# Genome-Wide Association Study Reveals a Genomic Region Associated with Mite-Recruitment Phenotypes in the Domesticated Grapevine (*Vitis vinifera*)

**DOI:** 10.3390/genes12071013

**Published:** 2021-06-30

**Authors:** Erika R. LaPlante, Margaret B. Fleming, Zoë Migicovsky, Marjorie Gail Weber

**Affiliations:** 1 Department of Plant Biology, Program in Ecology, Evolution, and Behavior, Michigan State University, East Lansing, MI 48824, USA; erl48@nau.edu (E.R.L.); flemi221@msu.edu (M.B.F.); 2 Department of Plant, Food, and Environmental Sciences, Faculty of Agriculture, Dalhousie University, Truro, NS B2N 5E3, Canada; Zoe.Migicovsky@dal.ca

**Keywords:** acarodomatia, defense mutualisms, indirect defense, mite domatia, mite defense, powdery mildew

## Abstract

Indirect defenses are plant phenotypes that reduce damage by attracting natural enemies of plant pests and pathogens to leaves. Despite their economic and ecological importance, few studies have investigated the genetic underpinnings of indirect defense phenotypes. Here, we present a genome-wide association study of five phenotypes previously determined to increase populations of beneficial (fungivorous and predacious) mites on grape leaves (genus *Vitis*): leaf bristles, leaf hairs, and the size, density, and depth of leaf domatia. Using a common garden genetic panel of 399 *V. vinifera* cultivars, we tested for genetic associations of these phenotypes using previously obtained genotyping data from the Vitis9kSNP array. We found one single nucleotide polymorphism (SNP) significantly associated with domatia density. This SNP (Chr5:1160194) is near two genes of interest: *Importin Alpha Isoform 1* (VIT_205s0077g01440), involved in downy mildew resistance, and *GATA Transcription Factor 8* (VIT_205s0077g01450), involved in leaf shape development. Our findings are among the first to examine the genomic regions associated with ecologically important plant traits that facilitate interactions with beneficial mites, and suggest promising candidate genes for breeding and genetic editing to increase naturally occurring predator-based defenses in grapevines.

## 1. Introduction

Plants have evolved a variety of phenotypes to defend themselves against herbivores and pathogens. While many of these defensive traits act directly on pests to reduce plant damage (deemed “direct defenses”), other traits (deemed “indirect defenses”) provide defense by increasing populations of arthropods that benefit the plant by consuming or deterring herbivores or pathogens [1,2]. Despite their prevalence and known ecological importance, the genetic drivers and constraints of most indirect defense phenotypes remain poorly understood compared to the more thoroughly studied direct defense traits [3].

Among the most notable plant structures that provide indirect defense to plants are acarodomatia (also called “mite domatia” and hereafter referred to as “domatia”). Domatia are small, morphogenetic structures on the undersides of leaves that recruit and retain beneficial mites that defend the plant by consuming herbivores and plant pathogens [4]. Occurring most commonly in the abaxial vein axils of woody plants, domatia generally take the form of small (usually <1 mm^3^) invaginations in the leaf lamina covered with a dense layer of trichomes, resulting in a covered chamber that provides shelter for beneficial mites and their eggs [5,6]. Beneficial mites thrive in the hospitable environment that domatia provide, which can protect mites and their eggs from desiccation and predation, and can provide food for mites by trapping pollen and spores [7,8,9]. In turn, mites consume herbivores and fungi found on the leaf surface, decreasing plant damage and increasing plant fitness in the right conditions [7,9,10]. Unlike galls, mite domatia are heritable features of plants, and do not require the presence of mites for their expression [7]. A large body of experimental research has demonstrated that heritable phenotypic variation in the presence, size, and density of domatia on plant leaves can impact the density of beneficial mite abundance on leaves and, in turn, the density of pest and pathogen loads [5,8,9,10,11,12,13,14].

Like mite domatia, trichomes on leaf lamina can increase the abundance of both predacious and fungivorous mites on plant leaves in some plant taxa [12,15]. In these systems, laminar trichomes provide protection (e.g., from predation or abiotic stress) or food (e.g., trapped pollen and spores) for beneficial mite populations on leaves [12,16,17]. Unlike mite domatia, which primarily function as defenses, laminar trichomes can provide a suite of functions on plant leaves. Leaf trichomes can alter the boundary layer, mediate gas exchange, and protect the leaf from harsh abiotic conditions such as UV radiation and cold temperatures [18]. Thus, while trichomes may function broadly to facilitate indirect defense across plants, their defensive role has only been tested for and demonstrated in a handful of systems. However, in these cases, trichomes on the leaf lamina can be considered indirect defensive traits, even if they serve multiple functions concurrently. 

Here, we investigate the genetic drivers of mite domatia and indirect defense-related trichomes in the cultivated grapevine, *Vitis vinifera*. The cultivated grape has considerable heritable variation in mite-recruitment phenotypes [7,19], and a large body of experimental research has established a firm link between these phenotypes and their role in mediating indirect defensive functions in grapes. In particular, the presence of leaf trichomes and the presence, size, and density of mite domatia impact the abundance of predatory mites on leaves (e.g., family Phytoseiidae) [13,15,16,19] and fungivorous mites (e.g., family Tydeidae) [9,11,20]. Increases in beneficial mite abundance in turn can lead to with decreased pest and pathogen loads on *Vitis* leaves, including decreased outbreaks of herbivorous spider mites (family Tetranychidae) [13,21], as well as the fungal pathogens of powdery mildew (*Uncinula necator*) and downy mildew (*Plasmopara viticola*) [7,9,11,20,22]. Given that mildew and spider mites are both top causes of damage in vineyards, where they can cause significant economic losses to growers [23,24], the genetic investigations of the phenotypes that mediate indirect defense interactions in grapes are of direct agricultural and economic importance. Further, genetic studies in cultivated grape are highly feasible, as the cultivated grape is a globally recognized model system with a fast-growing, publicly accessible genomic toolkit, including a genome assembly (487–500 Mbp genome; 2n = 38 chromosomes) [25,26,27,28].

We capitalize on the extensive phenotypic variation in *V. vinifera* for beneficial mite-related phenotypes, the large body of ecological research tying these phenotypes to mite defense, and the *Vitis* genomic toolkit to conduct a genome-wide association study (GWAS) for mite indirect defense phenotypes. Specifically, we use a pre-existing common garden diversity panel of 399 *V. vinifera* cultivars and publicly available Vitis9kSNP array data to test for single nucleotide polymorphisms (SNPs) associated with five mite-related phenotypes: three measures of domatia (size, density, and depth) and two measures of leaf trichomes (presence/absence of bristles and hairs). Our goal is to identify candidate genetic regions underlying key phenotypes that are tied to the recruitment and retention of beneficial mites on grapevine leaves, and to provide insight into the genetic control of mite defense mutualisms across plants more generally.

## 2. Materials and Methods

### 2.1. Association Panel

Plants used in this study were located in a common garden grapevine diversity panel of *V. vinifera* accessions maintained as part of the US Department of Agriculture (USDA) Grape Germplasm Repository at Wolfskill Farms in Winters, Canada. Information on the geographic origins of the accessions can be found in Table S1 of [25]. Pest management practices at this location consisted of spraying with sulfur every two weeks. 

We used genotyping data from [25], wherein the 950 *V. vinifera* accessions in the USDA collection were sequenced with the Vitis9kSNP array [29]. Using the entire genotyping dataset, we imputed missing SNPs with LinkImpute, optimized with k = 7 and l = 51, to obtain an accuracy of 0.93 [30]. The imputed dataset was filtered for accessions with no clonal relationships to any other accessions in the USDA germplasm collection (*n* = 399, as identified in [25]). The minor allele frequency was set to 0.01 in Tassel Version 5 [31], resulting in 4523 SNPs for downstream analyses.

### 2.2. Phenotyping

To quantify phenotypic variation in mite-related traits across the panel, we collected three mid-shoot, fully expanded adult leaves from one individual per cultivar for each of 399 accessions uniquely represented in the USDA collection (1197 leaves total) in June of 2018. All collected leaves occurred beyond the sixth node from the base of the shoot coming from the previous year’s wood, in accordance with [19]. 

**Figure 1:**
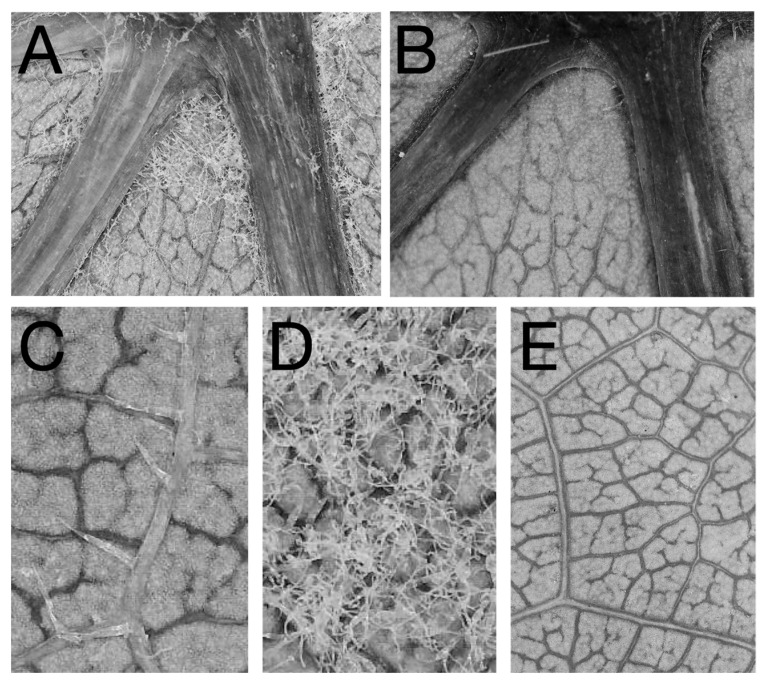
Photographs of phenotypes evaluated in the common garden study: (**A**) a relatively large, dense mite domatium. (**B**) A vein axil of a cultivar that lacked mite domatia. Cultivars ranged from having (**C**) leaf bristles and (**D**) leaf hairs present to those with glabrous abaxial leaf surfaces (**E**). Samples used for photographs were from accessions (from top left to bottom right) V.2510, V.0409.3, V.2167.1, V.0432.1, and V. 0416.1.

**Figure 2:**
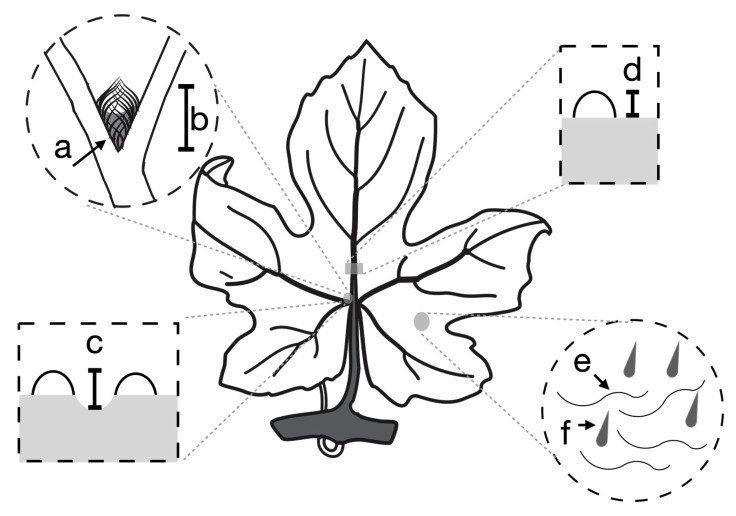
The location of scored phenotypes on the grape leaf. Domatia density (**a**) and size (**b**) were measured on the most basal primary vein axil on the left abaxial side of the leaf. Pit depth was measured as the difference between the height of the most basal primary vein axil on the left abaxial side of the leaf (**c**) and the height of the main vein above the leaf lamina halfway between the primary and secondary axils (**d**). The presence or absence of leaf hairs (**e**) and bristles (**f**) was measured on the abaxial leaf surface.

For each leaf, we measured five traits: domatia size, domatia density, domatia pit depth, leaf bristles, and leaf hairs (Table 1, Figure 1 and Figure 2). These traits were selected based on evidence in the literature associating their morphology with increased abundance of beneficial (predacious and fungivorous) mites leading to reductions in pests and pathogens (spider mites and powdery mildew) (Table 1). All three domatia traits (density, size, and pit depth) were scored on the domatium located to the left of the midvein at the most basal vein axil, in accordance with [32,33] (Figure 2). Domatia density was measured as the density of trichomes in the domatium with a ranking of 0 (no hairs) to 9 (very dense) according to the OIV codes O-084, U-33 and O-085, U-34, a standard rating system used by grape breeders [33] (Figure 2a). Domatia size was characterized as the distance between the point where veins intersected and the point on the leaf blade where domatium trichome density was reduced (Figure 2b), in accordance with [7,9,11]. Domatia pit depth refers to the degree of invagination of the lamina inside the domatium, which reflects the size of the cavern available for mites to occupy. Pit depth was measured by subtracting the height of the midvein midway up the leaf (Figure 2d) from the height of the midvein at the vein axil (Figure 2c). The presence or absence of two types of leaf trichome was scored according to binary traits: leaf hairs (Figure 1c and Figure 2e), ribbon-like, prostrate trichomes that are slender, flattened, and approximately 2,000 μm long [corresponding to OIV code 084-1 (U-33 UPOV)], and leaf bristles (Figure 1d and Figure 2f), shorter, unbranched, conical, erect trichomes roughly 300 μm long [corresponding to OIV code 085-1 (U-34)] [33,34,35]. All traits were scored on dried leaves, with the exception of domatia pit depth, which was scored on fresh leaves to avoid errors introduced by vein depth shrinking when dried.

Genotype averages were calculated for each trait and used in all downstream analyses. Correlations between phenotypes were tested using a Pearson’s correlation matrix with Holm corrected *p*-values for multiple comparisons [36] using the corr.test function in the R package psych [37]. Correlation matrix results were visualized using the corrplot.mixed function in the R package corrplot [38] and the ggpairs function in the GGally package [39]. All analyses were run using R version 4.0.3 [40].

### 2.3. Genome-wide Association Study Analysis (GWAS) and Genomic Prediction

We conducted a GWAS for each trait independently using the GAPIT R package (Version 3) [41]. Model selection within GAPIT found that, for all traits, the optimal number of principal components to include in the model was zero. Kinship was calculated within GAPIT using the VanRaden method. We tested for significant genetic associations with phenotypes using a mixed linear model. QQ plots indicated that this model adequately accounted for relatedness (Figure S1). Genes within 10 kb (the average window of linkage disequilibrium in *Vitis* [25,29]) and 75 kb (a less conservative window to identify potentially interesting, but less likely, candidates) of the significant SNP were identified in the Vvinifera_457_v2.1 genome [42] obtained from data.inrae.fr (doi:10.15454/1.5009072354498936E12) on 5 May 2021.

In addition to GWAS, we performed genomic prediction in R using the PopVar package [43]. The specific parameters selected included the rrBLUP model, no further filtering based on minor allele frequency, and a fivefold cross-validation procedure, which was replicated three times. The results of this analysis were visualized using the ggplot2 R package [44].

## 3. Results

### 3.1. Phenotyping

**Figure 3:**
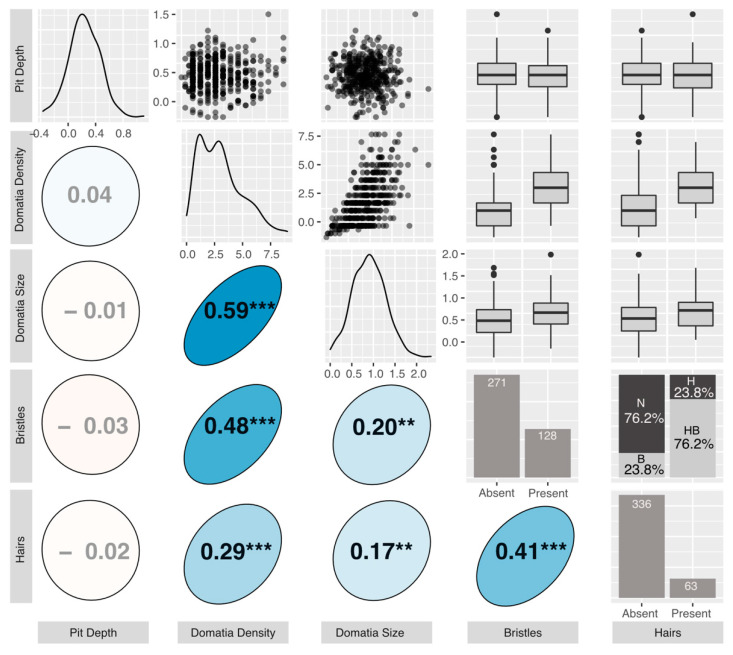
The graphical display of the correlation matrix for the measured phenotypes. All phenotypes displayed are accession means: *Upper triangle:* plotted relationships of the measured phenotypes. For box and whisker plots, the box represents the 25th and 75th percentiles, the bar represents the median, and dots are outliers beyond the quartiles. N = neither hairs nor bristles, B = bristles only, H = hairs only, HB = hairs and bristles. *Lower triangle:* Pearson’s correlation coefficients. Positive correlations are displayed in blue and negative correlations in red. Color intensity and the size of the circle are proportional to the correlation coefficients. Stars denote “holm” corrected *p*-values (*** < 0.001, ** < 0.01). *Diagonal:* density distributions of the phenotypes.

We found considerable variation in mite-recruitment phenotypes across our common garden genotypes (Figure 3). Average domatium size ranged from 0.00–2.33 μm (mean = 0.90 μm) and was positively correlated with average density rating, which ranged from 0 to 9, with a mean of 3.09 (*r* = 0.59, *p* < 0.001). Average pit depth ranged from −0.37 (negative depths represent cases where the laminar vein axil tissue was less depressed than the laminar tissue halfway up the midvein) to 1.10 μm (mean = 0.23 μm), and was not correlated with any other measured phenotypes (Figure 3). The presence or absence of bristles or hairs was always consistent across replicates of the same genotype. Of the 399 genotypes, 80 had bristles-only, 15 had hairs-only, and 48 had both bristles and hairs. Bristles and hairs were positively correlated with one another (*r* = 0.41, *p* < 0.001), with domatia size (bristles: *r* = 0.2, *p* < 0.01; hairs: *r* = 0.17, *p* < 0.01) and domatia density (bristles: *r* = 0.48, *p* < 0.001; hairs: *r* = 0.29, *p* < 0.001) (Figure 3).

### 3.2. GWAS Results

**Figure 4:**
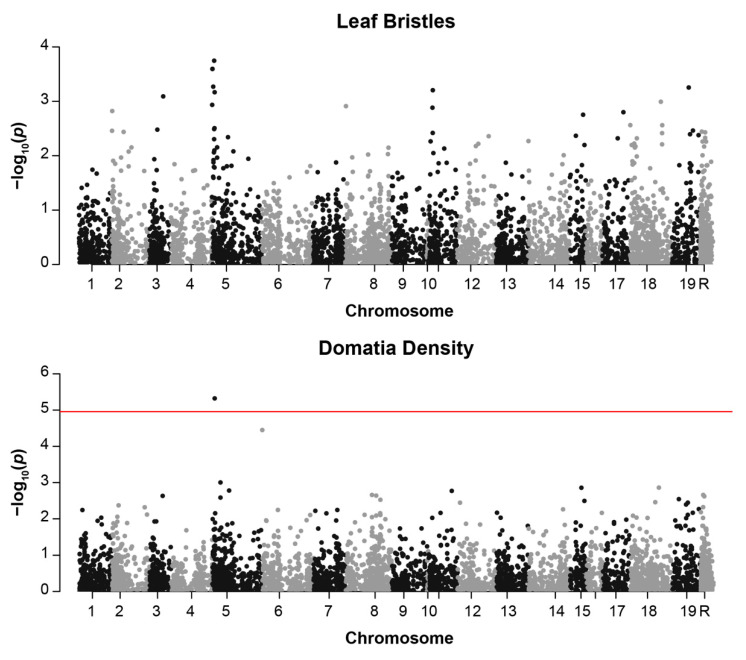
Manhattan plots displaying genome-wide association results for: (**top**) bristle presence/absence and (**bottom**) domatia density. Plots are based on a MLM model with minor allele frequency of >1%. The red line indicates the Bonferroni-corrected threshold for significance with α = 0.05. SNPs on chromosome R are found on contigs that are not anchored to the reference genome.

Only one of the five measured phenotypes, domatia density, was significantly associated with an SNP in our analysis. A single SNP on chromosome 5, 1160194, was significantly associated with domatia density with a Bonferroni-corrected α of 0.05/4539 = 1.1 × 10^−5^ (*p* = 4.69 × 10^−6^, *r^2^* = 0.35, Table 2, Table S2). This same SNP displayed a non-significant trend toward an association with bristle presence/absence (*p* = 0.00018, *r^2^* = 0.34) (Figure 4 and Figure S2, Table S1). The associated SNP on chromosome 5 is flanked by two genes of interest: *GATA Transcription Factor 8* (VIT_205s0077g01450) [45] and *Importin Alpha Isoform 1* (VIT_205s0077g01440) [46]. Two additional genes with intriguing functional annotations were also identified within 75 kb. The first, an ortholog of *Powdery Mildew Resistance 5* (VIT_205s0077g01340), is an O-acetyltransferase involved in powdery mildew resistance [47], located about 74 kb upstream of the SNP. The second, *Glabrous Inflorescence 2* (VIT_205s0077g01390) [48], is a zinc finger protein that regulates trichome development, located about 31 kb upstream of the SNP.

### 3.3. Genomic Prediction Results

**Figure 5:**
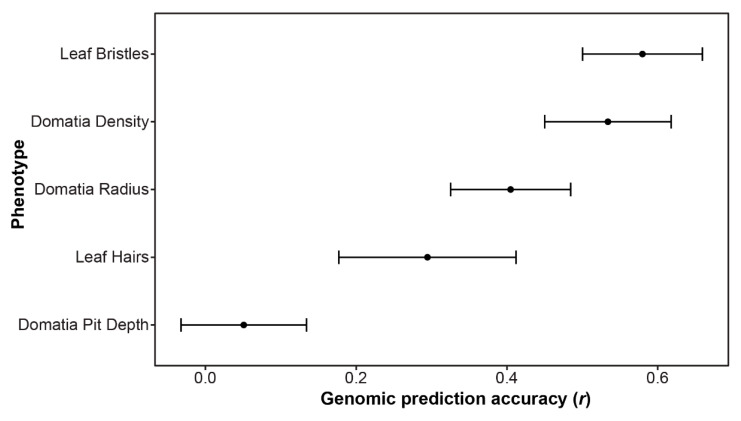
Genomic prediction accuracy (*r*), indicating the correlation between the observed and prediction phenotype, as estimated using an rrBLUP model with fivefold cross-validation, repeated three times. The average *r* ± the standard deviation is plotted.

In cases where no significant SNPs were identified using GWAS, it is possible that the trait is controlled by numerous small effect loci throughout the genome. In such instances, genomic prediction can provide insight into the genetic architecture of the trait. Estimating the genomic prediction accuracy (*r*) across the five mite-recruitment phenotypes measured in this study resulted in values ranging from 0.05 for domatia pit depth to 0.58 for leaf bristles. The second highest accuracy was 0.53 for domatia density (Figure 5).

## 4. Discussion

Understanding the genetic underpinnings of plant defense phenotypes is of broad economic and ecological importance. However, indirect defensive traits—plant phenotypes that provide protection by attracting enemies of herbivores and pathogens to leaves—have received little attention in genomic studies. Here, we conducted a genome-wide association study of five phenotypes known to provide indirect defense to grape plants via the attraction of beneficial (predacious and fungivorous) mites. We observed considerable variation in mite-recruitment phenotypes among the 399 cultivars we measured. Mite-recruitment phenotypes were correlated across cultivars, suggesting that suites of indirect defense traits may evolve and function together in *V. vinifera*. The phenotypic variation displayed within this species was sufficient for genetic association tests. The most notable finding of the GWAS analysis was the identification of a single SNP on chromosome 5 (Chr5:1160194) that was significantly associated with domatia density. Given that domatia are primarily composed of bristles in *Vitis*, and that domatia density and bristle presence are also correlated in this as well as other studies [19,49], it is not surprising that the same SNP was also trending toward association with the presence of laminar bristles. The two genes flanking this SNP in the 10kb window of LD should be considered as candidates in future studies.

We found two promising candidate genes within 10 kb of the SNP associated with domatia density on chromosome 5. The gene directly downstream of the SNP, *VvGATA8* (VIT_205s0077g01450), encodes a short B-GATA transcription factor with a C-terminal Leucine–Leucine–Methionine (LLM) domain. In the closest *Arabidopsis* homolog, *AtGATA16*, this domain has been shown to control leaf shape, as GATA overexpressors with a mutated LLM domain have rounder leaves with shorter petioles [50]. GATA transcription factors are also involved in other developmental processes in *Arabidopsis*, including greening, cytokinin sensing, and phyllotaxy. Mutants with reduced expression of *AtGATA16* showed increased number of branches and a smaller angle between the branch and the stem [51]. The GATA gene family was duplicated after the divergence of *Vitis* and *Arabidopsis*, providing opportunities for neofunctionalization of GATA genes in *Vitis*. Interestingly, leaf shape and trichome density are genetically linked in Vitis [49]. Additionally, because leaf shape is tied to vein patterning and domatia occur in vein axils, leaf shape and domatia development may be controlled by the same genes or pathways.

The second candidate gene within the 10 kb window of the significant SNP associated with domatia density was VIT_205s0077g01440. This gene is directly upstream of the significant SNP and encodes *Importin Alpha Isoform 1*, which is involved in nuclear import of plant immune response-related proteins, as well as pathogen effector proteins [46]. In *Vitis*, Importin alpha 1 specifically imports the *Plasmopara viticola* (downy mildew) effector protein PvAVH53 to the nucleus [46]. Genetic interactions between domatia, which are a structural feature of the leaf epidermis, and susceptibility to a disease mediated by the leaf epidermis are both plausible, as selection for mite domatia may be correlated with selection for or against genes controlling downy mildew resistance. Furthermore, the predatory mite *Tydeus caudatus* (now *Tenuipalpus caudatus*) consumes downy mildew as an alternate food source [22]. This mite is found on the undersides of leaves of domatia-bearing plants such as *Viburnum tinus* and several *Vitis* species [52]. Further work is needed to uncover the exact connections between downy mildew susceptibility and mite-recruitment phenotypes. 

While the genes within the 10 kb window of the significant SNP identified in our study are the most likely candidates for involvement in domatia control due to the rate of LD decay in *Vitis*, there are several genes within 75 kb of the SNP that also deserve brief discussion. For example, a study by Barba et al. [19] using *Vitis* hybrids revealed a QTL on chromosome 5 associated with leaf hairs which contained a promising candidate gene, *Glabrous Inflorescence Stems 2* (VIT_205s0077g01390), which is about 31 kb from the SNP our study identified. It encodes a zinc finger protein that integrates cytokinin and gibberellic acid signaling to regulate trichome development [48]. Additionally, a final gene, *Powdery Mildew Resistance 5* (VIT_205s0077g01340), was about 74 kb from the SNP our study identified. Although this gene is possibly too far away to be in LD with this SNP, the well-established relationships between domatia, trichomes, mites, and powdery mildew [7,9,11,20] make it worth further consideration in future studies. This gene encodes an O-acetyltransferase active in pectin formation during cell wall development. Arabidopsis loss-of-function mutants have smaller rosettes and epidermal cells due to reduced cell expansion, and their cell walls have different pectin composition [47]. This gene confers resistance to multiple species of powdery mildew but no other diseases.

In contrast to GWAS, which examines the correlation between individual markers and a trait of interest, genomic prediction uses all markers to predict the phenotype. Genomic prediction is therefore a valuable tool for the prediction of complex, polygenic traits. Similar to the GWAS results, leaf bristles and domatia density had the most promising results for genomic prediction in this study. A previous study which included many of the same grapevine accessions examined here, as well as the same SNP array, estimated genomic prediction accuracies (*r*) ranging from 0.1 to 0.76 [28]. Traits such as berry firmness (*r* = 0.58) and bloom date (*r* = 0.54) had genomic prediction accuracies similar to leaf bristles (*r* = 0.58) and domatia density (*r* = 0.53) [28]. Previous work on apple polyphenols found genomic prediction accuracies ranging from below 0 (not predictable) to 0.49 ([53]), while another study exploring numerous agriculturally important phenotypes in apples found the highest prediction was 0.57 for harvest season, followed by measurements of fruit width (*r* = 0.48) and length (*r* = 0.47) [54]. These findings suggest that, even in the absence of numerous significant SNPs for GWAS, there is a strong genetic basis to the mite-recruitment traits examined in our study.

We also found that domatia and leaf trichome traits were broadly correlated in our study, suggesting a suite of mite-recruitment traits that co-vary across domesticated grapes. Domatia and trichome traits have also been found to correlate in *Vitis* in other studies [16,19,34]. Interestingly, a study by Barba et al. [19] using *Vitis* hybrids revealed a QTL on chromosome 1 that explained most of the phenotypic variance in leaf domatia, hairs, and bristles, suggesting that these phenotypes may be controlled by shared genetic regions in this group. Suites of defensive traits are hypothesized to be under selection to coevolve if they work together to facilitate defensive interactions [55,56]. Future work aimed at understanding the genetic and selective drivers of indirect defense trait correlations in *Vitis* could provide a promising first test of plant defense syndromes in mite defensive traits.

Our study is the first GWAS to focus on beneficial mite-recruitment phenotypes in *V. vinifera*, and it complements previous work using QTL approaches to examine similar traits in *V. vinifera* and *Vitis* hybrids (e.g., [19,57,58]). GWAS and QTL studies are both useful approaches for determining the relationship between genetic markers and a trait of interest. However, in contrast to QTL mapping, which performs genetic mapping using a biparental cross, GWAS makes use of a diverse population. Mapping resolution is improved when performing GWAS due to the increased recombination rate among individuals. At the same time, rare variants which fall below the minor allele frequency threshold will be missed by GWAS. Future work could build on this collective body of research in several ways. First, more comprehensive GWAS panels with higher coverage (>9k SNPs) could reveal additional associations missed in this study. Second, work that incorporates phenotypic variation across space and time would build on the single-location, single-time point limitations of the work presented here. Third, using additional genomic/transcriptomic tools to confirm the importance of the candidate genes identified in this and other studies will solidify the potential link between genotype and phenotype. Finally, pairing genetic manipulations with field experiments will allow for tests of functional links between the candidate genes identified here, the measured phenotypes, and indirect defense via mite recruitment.

## 5. Conclusions

Phenotypes that recruit predacious and fungivorous mites to grape leaves have been well studied for their role in defending plants from damaging pathogens (such as powdery mildew) and small herbivores (such as spider mites). Our analysis of five beneficial mite-recruitment phenotypes across 399 grape cultivars revealed notable genetic associations and interesting candidate genes. Most notably, domatia density displayed a strong association with a SNP on chromosome 5, close to several potential candidate genes. Given the considerable economic toll of powdery mildew and spider mites in grape and wine production worldwide [23,24], this study presents an exciting step in understanding the genetic underpinnings of phenotypes that provide defense in grapevines without the use of harmful pesticides or fungicides. Furthermore, mite domatia have evolved repeatedly many times across plants, occurring in over 2000 plant species from over 20 plant families, including in other important forestry and crop plants such as oak, maple, beech, cherry, and coffee [4,59]. Thus, this study demonstrates its importance as one of the first to examine the genetic associations of these widespread indirect defensive traits.

## Figures and Tables

**Table 1:** The phenotypes used in this study and associated citations supporting their role in mediating beneficial mite populations. All cited papers present research conducted on *Vitis*, with the exception of [8], which is focused on *Cinnamomum camphor*. Additional details on measurements can be found in the methods.

Trait	Description	Citations Demonstrating Trait Enhances Beneficial Mite Abundance
Domatia size	The size of the most basal domatium just left of the midvein	[7,11]
Domatia density	The density of trichomes making up the most basal domatium just left of the midvein	[7,13]
Domatia pit depth	The degree to which the leaf lamina is depressed inside the domatia, creating a cavern for mites to occupy	[8]
Leaf bristles	The presence or absence of erect trichomes on the abaxial leaf lamina	[16,17,19,22]
Leaf hairs	The presence or absence of prostrate, ribbon-like trichomes on the abaxial leaf lamina	[16,17,19,22]

**Table 2:** Genes of interest and their locations relative to the SNP significantly associated with domatia density. The coordinates for each gene and the SNP of interest are listed sequentially. Genes within ±10 kb of the SNP are in bold.

Gene ID v.2	Annotation	Coordinates	Gene ID v.0	Arabidopsis Homolog
VIT_205s0077g01340	*Powdery Mildew Resistance 5*	1085706-1087504	GSVIVG01035039001	At5g49340
VIT_205s0077g01390	*Glabrous Inflorescence 2*	1128866-1129489	Not present	At1g67030
VIT_205s0077g01440	*Importin Alpha Isoform 1*	1148995-1153335	GSVIVG01035047001	At3g06720
SNP Chr5:1160194		1160194		
VIT_205s0077g01450	*GATA Transcription Factor 8*	1163455-1164868	GSVIVG01035048001	At3g06740

## Data Availability

All data are available in the Supplemental Materials.

## Supplementary Materials

The following are available online at https://www.mdpi.com/article/10.3390/genes12071013/s1, Figure S1: QQ plots for the five mite-recruitment phenotypes; Figure S2: Manhattan plots for domatia size, depth, and leaf hairs, Table S1: Phenotype Data, Table S2: GWAS results tables.
