# Supplementary material for: Genome-Wide Association Study Reveals a Genomic Region Associated with Mite-Recruitment Phenotypes in the Domesticated Grapevine (Vitis vinifera)
Source: Genes (Basel). 2021 Jun 30;12(7):1013. doi: 10.3390/genes12071013 (PMC8307218; doi:10.3390/genes12071013)
Supplement: Supplementary file 1 [file genes-12-01013-s001.zip › Figures_S1_S2.pdf]

Supplementary data to: **Genome-wide association study reveals genomic region associated with beneficial mite recruitment phenotypes in the domesticated grapevine (*Vitis vinifera*)**

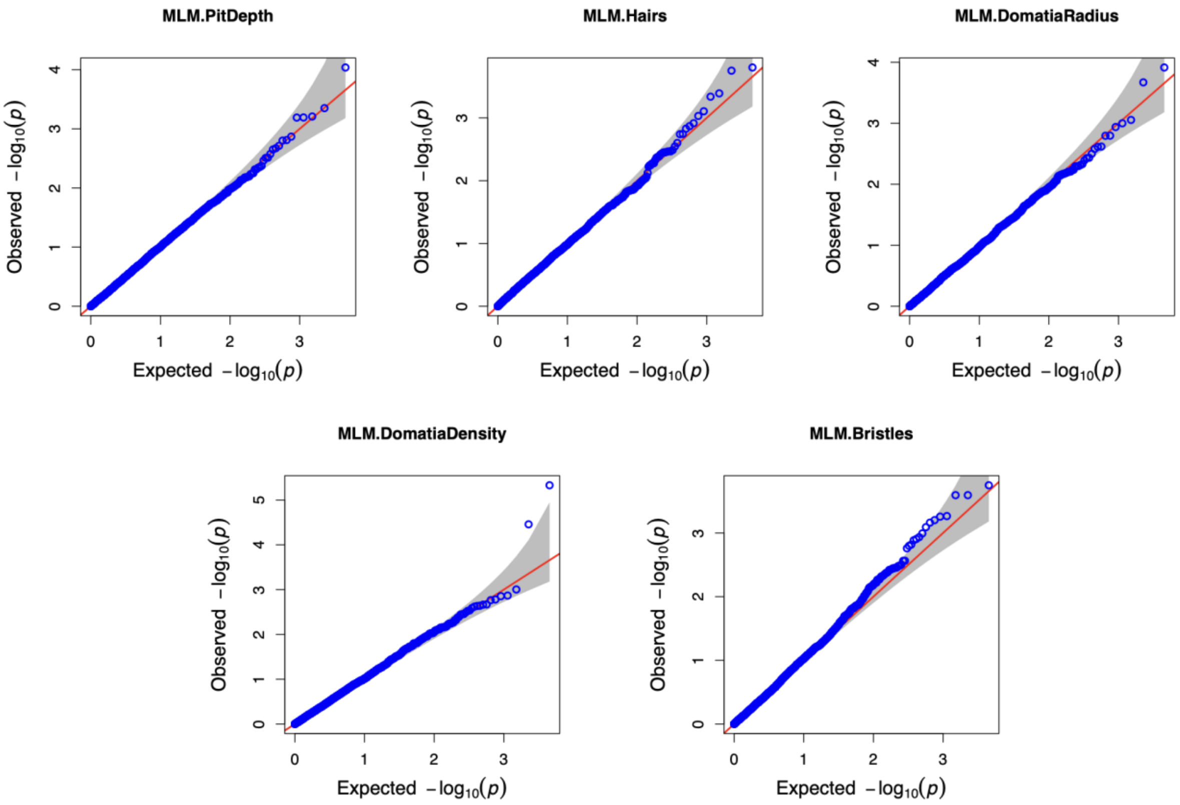

Figure S1: QQ plots for the five mite-recruitment phenotypes.

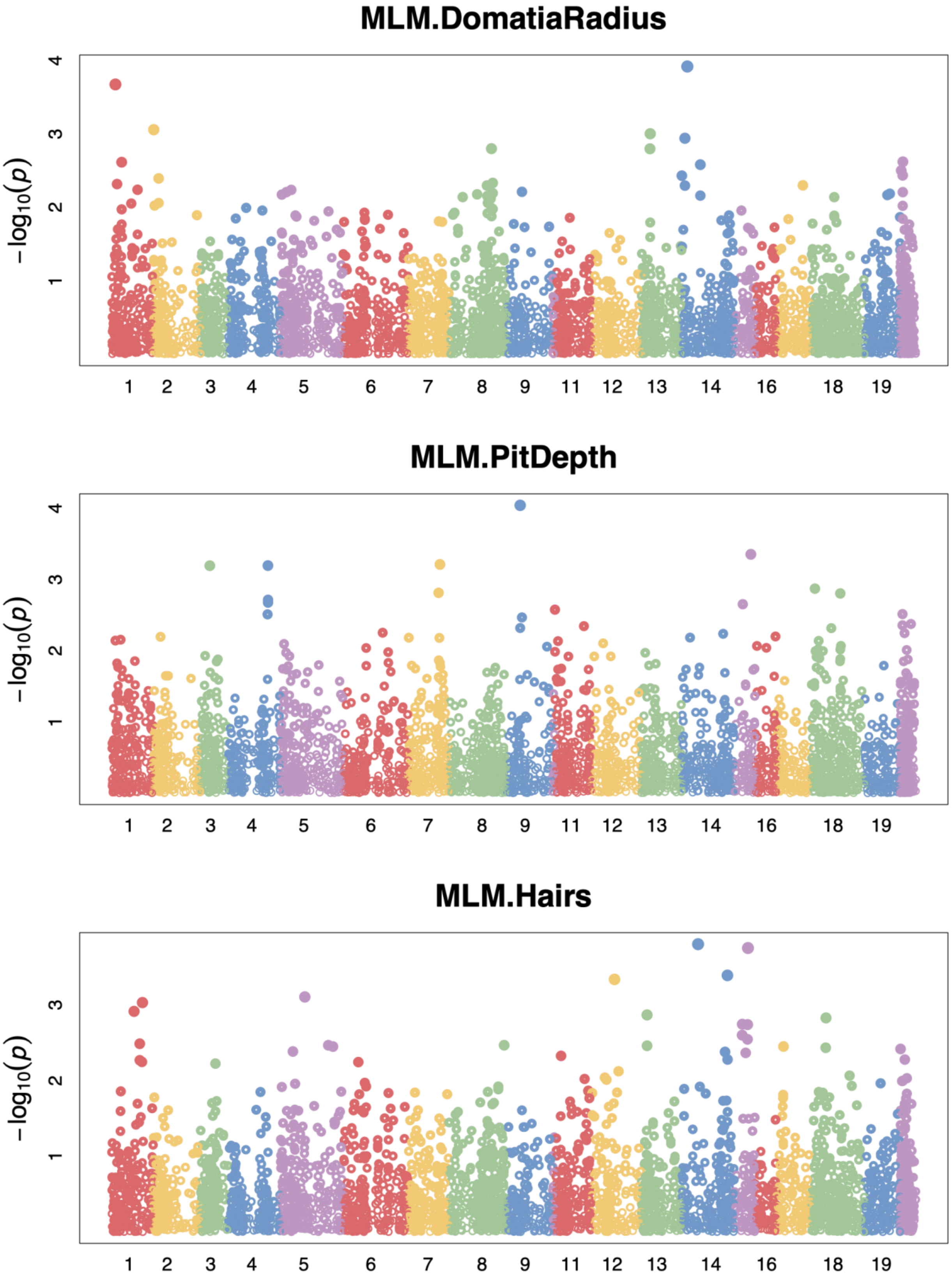

Figure S2: Manhattan plots for domatia size, depth, and leaf hairs.
